# Development of a secure, standardised and interoperable surveillance platform for race-related injury and illness data within the UCI men’s and women’s road cycling world tour: a study protocol

**DOI:** 10.1136/bmjsem-2025-003192

**Published:** 2026-02-19

**Authors:** Thomas Fallon, Robbe Decorte, Steven Verstockt, Debbie Palmer, Xavier Bigard, Neil Heron

**Affiliations:** 1 Centre for Public Health, Queen’s University Belfast, Belfast, UK; 2 Sports Medicine Research Centre, University of Edinburgh; 3 ELIS Department, Ghent University, Ghent, Belgium; 4 Edinburgh Sports Medicine Research Network, Institute for Sport, PE and Health Sciences, University of Edinburgh, Edinbugh, UK; 5 Union Cycliste Internationale, Aigle, Switzerland; 6 Queen’s University Belfast, Belfast, Belfast, UK; 7 Keele University, Keele, UK

**Keywords:** Epidemiology, Cycling, Surveillance, Injuries

## Abstract

**Background:**

Professional road cycling is associated with a high incidence of traumatic injuries. Despite these risks, current injury data-collection methods lack consistency and standardisation, thereby limiting meaningful surveillance and prevention efforts.

**Aim:**

To describe the development of a secure, centralised injury surveillance system for elite cycling that enables standardised data collection, contextual integration and long-term injury tracking while ensuring compliance with ethical and data protection standards.

**Methods:**

The system integrates an incident-activated Qualtrics-based injury reporting platform (hosted at Queen’s University Belfast) used by team medical staff and accessed via (or) within a secure, access-controlled server infrastructure hosted at IDLab, Ghent University. The database is protected by role-based authentication, encrypted data transmission and application programming interface-based access controls. Race footage and contextual data (eg, weather including ambient temperature, terrain) will be linked to medical reports to improve understanding of injury mechanisms.

**Ethics and governance:**

The system is designed to comply with the General Data Protection Regulation. Data pseudonymisation, consent protocols and ethics are built into the design. All access is logged, monitored and restricted to authorised users only.

**Expected outcomes:**

The project is expected to improve the quality and completeness of injury data in professional road cycling, facilitate epidemiological research, inform the development and evaluation of injury prevention strategies and support international policy development.

**WHAT IS ALREADY KNOWN ON THIS TOPIC**

- Elite road cycling carries a high risk of injury due to high speeds, dense pelotons and variable environmental conditions.
- Existing injury and illness surveillance systems in cycling are lacking and inconsistent, and when they do exist, they are often limited to team-level data.
- Other major sports (eg, football, rugby) have centralised injury surveillance frameworks that have successfully informed rule and policy changes to reduce injury risk.

**WHAT THIS STUDY ADDS**

- This study outlines the development of the first secure, standardised and interoperable injury and illness surveillance system for Union Cycliste Internationale (UCI) World Tour cycling.
- The system integrates medical reporting, contextual race data and automated data transfer through a JSON Web Token-authenticated application programming interface, aligned with International Olympic Committee consensus definitions.
- This study outlines the development of the first secure, standardised and interoperable injury and illness surveillance system for UCI World Tour cycling.

**HOW THIS STUDY MIGHT AFFECT RESEARCH, PRACTICE OR POLICY**

- Enables longitudinal epidemiological research to identify mechanisms, risk factors and the types, locations and severities of cycling injuries.
- Informs UCI and SafeR policy decisions on race safety, equipment standards and rider health management.
- Informs UCI and SafeR policy decisions on race safety, equipment standards and rider health management.

## Introduction

 Elite road cycling represents one of the most globally visible disciplines within competitive sport.^1^ However, it also remains among the most hazardous, with injuries frequently sustained during competition due to high velocities, technical descents, dense pelotons and unpredictable environmental conditions.^2^ Despite this elevated risk profile, there is currently no standardised injury or illness surveillance framework in place for this cycling discipline.^3^

Injury surveillance systems provide structured frameworks for systematically capturing, managing and analysing sports-related injury data. However, these data are only as good as the methodology underpinning their collection. Consensus statements guide injury surveillance methodologies to ensure methodological homogeneity.^4^ The International Olympic Committee (IOC) first presented its consensus statement on injury and illness reporting in 2020.^5^ However, many sports had consensus statements to guide injury and illness surveillance before the IOC consensus was published. Such as football in 2006,^6^ rugby union in 2007,^7^ multisport events in 2008,^8^ rugby league^9^ and tennis^10^ in 2009, horse racing in 2012,^11^ athletics sports in 2014,^12^ aquatic sports^13^ and cricket^14^ in 2016,^13^ mass events in 2019,^15^ golf in 2020.^16^

Since the publication of the IOC multisport consensus in 2020, many sports have updated their consensus statements to align with this. To date, over 30 consensus statements are represented on the IOC website.^17^ These range from football,^18^ tennis,^18^ snow sports,^19^ circus^20^ and artistic gymnastics^21^ to population-specific groups such as para-athletes,^22^ youth sport athletes^23^ and female athletes,^24^ environment-specific^25^ and illness-specific.^26 27^

**Figure 1:**
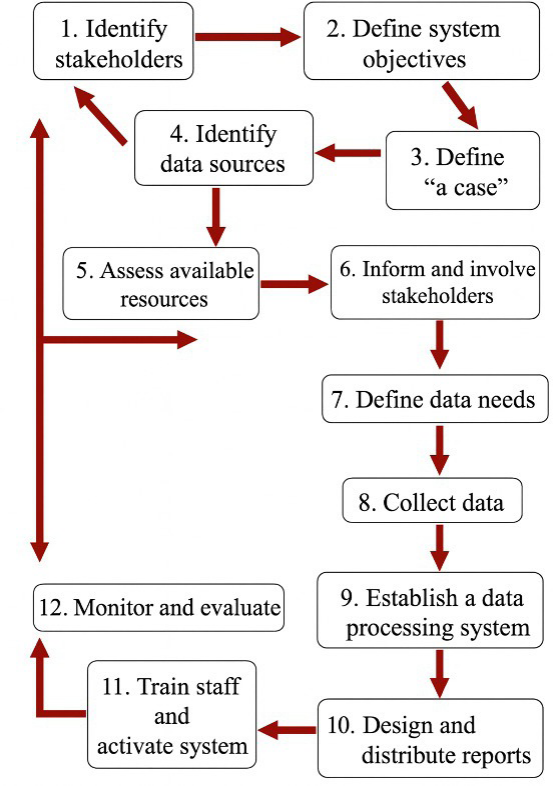
WHO steps to designing and building a surveillance system.^32^

The first IOC statement extension for competitive cycling was published in 2021^28^; however, since then, only three studies have aligned with its recommendations.^2 29 30^ With the range of consensus statements on the reporting of injuries and illness across sports, populations and conditions, surveillance systems provide us with fundamental structures to onboard this information. The WHO guidelines outline how surveillance systems should define clear objectives, standardise case definitions, identify target populations, implement secure and systematic data collection and management processes and ensure rigorous ethical governance, analysis and reporting^31^ (figure 1, online supplemental material 1). Indeed, centralised surveillance systems have successfully informed safety interventions in many sports such as rugby and football, and have established centralised injury surveillance systems that have directly informed policy changes, such as modifications to tackle height^32^,^34^ or heading protocols,^35 36^ resulting in measurable reductions in injury risk.^37^,^45^ Yet cycling lacks an equivalent infrastructure.

The Union Cycliste Internationale (UCI) Agenda 2030 recognises this need, setting out the objective to ‘promote and support research in cycling epidemiology and medicine’ and to ‘establish an epidemiological database of medical and biological fitness criteria’.^46^ In alignment with these objectives, the development of a secure, standardised and interoperable platform capable of capturing and managing injury and incident data across teams and events within the UCI World Tour is a fundamental starting point. Such a system would strengthen epidemiological insight, foster a culture of safety and accountability across all levels of the sport, and inform evidence-based protection and safety measures.^47^

Existing data collection practices in cycling are limited, typically relying on team-level documentation, event-level or voluntary reporting, all of which vary in accuracy, completeness and methodological consistency.^2^ Consequently, these systems provide only a limited and inconsistent understanding of injury patterns and mechanisms. The absence of an equivalent epidemiological infrastructure within cycling represents a critical gap that limits both clinical response and long-term research into injury prevention and athlete health management.^47^

The UCI World Tour is the highest level of professional road cycling, comprising 18 men’s teams with a combined roster of 509 registered riders. Additionally, there are 16 men’s pro teams, which also guest compete on this world tour calendar. These athletes compete for 170 days across 35 events staged in 13 countries and on 4 continents. Correspondingly, the UCI Women’s World Tour comprises 14 professional teams, in addition to the 7 pro teams that compete on this calendar. The Women’s World Tour features 258 riders competing across 74 race days in 27 events spanning 12 countries and 3 continents. This global structure underscores the extensive geographical reach and competitive scope of elite professional road cycling.

This protocol outlines the structure of adding an interoperable injury surveillance log and expanding the current database to create an injury surveillance system. The overarching aim of this project is to develop, implement and evaluate the first secure, standardised and interoperable injury surveillance system for world tour road cycling. Specifically, the protocol aims to:

1. Establish a unified surveillance infrastructure for UCI World Tour and UCI Women’s World Tour that enables consistent, high-quality injury and illness data collection across teams and events.
2. Integrate incident-activated medical reporting with contextual race data, including terrain, weather and race dynamics, to improve understanding of injury mechanisms and situational risk factors.
3. Implement General Data Protection Regulation (GDPR)-compliant data governance, including pseudonymisation, secure server architecture, role-based access, application programming interface (API)-controlled data transfer and a complete audit trail.
4. Enable longitudinal epidemiological monitoring by linking repeated injuries, illnesses and race incidents across seasons at the individual rider level through privacy-preserving methods.
5. Provide a scalable model that supports:
  - Evidence-based injury prevention strategies.
  - Informed policy development for UCI and SafeR.
  - Future research into mechanisms of injury, risk profiling and emerging safety trends.
6. Lay the foundation for a centralised cycling-wide database that aligns with international consensus methodologies, enhances cross-disciplinary comparability and contributes to global sport-specific epidemiology.

## Database development

In response to collective safety concerns and Agenda 2030 priorities, the UCI Race Incident Database was developed by IDLab (Ghent University–imec) in collaboration with the UCI. Initially conceived as a central repository for reporting race crashes and accidents (referred to as ‘incidents’), the database addressed long-standing barriers related to fragmented data capture and accessibility. Over time, it has evolved into a key reference tool for governing and advisory bodies, including SafeR, a specialist independent entity focused on improving the safety of road cycling. It provides an empirical foundation for evidence-based recommendations and safety interventions within professional cycling.

The database and its management application enable efficient, real-time incident monitoring. Races are automatically linked to the UCI’s DataRide platform, while major (multi-rider) incidents are populated through automated analysis of verified media and social platforms (eg, X, formerly Twitter). Race commissaires and UCI stakeholders subsequently validate, refine and expand these entries to ensure a comprehensive and accurate record of each incident.

The work presented here builds on the UCI Agenda 2030, operationalising its objectives by proposing a structured framework to expand the UCI Race Incident Database to incorporate a comprehensive injury surveillance component. This injury log follows a format similar to that developed by the US Olympic and Paralympic Committee,^41^ aligning with the IOC consensus statement^5^ and its extension for cycling injuries.^29^ The protocol outlined herein has been informed by previous cycling injury and illness studies that used API-based and centralised systems within individual teams to integrate race data with epidemiological insight.^2 48^ Additionally, within-competition injury surveillance studies at the team and event level, which have previously been conducted in both downhill mountain biking^31^ and cyclocross, employ a previously published within-competition protocol using the same injury surveillance platform.^49^ By integrating the methodological insights and practical approaches, this protocol represents a critical step toward establishing a global, evidence-based infrastructure for injury surveillance within cycling.

The proposed system has the following primary objectives:

1. To develop a secure, GDPR-compliant, centralised platform for recording injuries sustained during UCI World Tour events.
2. To ensure standardisation in injury definitions and reporting procedures across participating teams in the World Tour and Women’s World Tour.
3. To integrate injury data with contextual information, such as race footage and environmental data.
4. To facilitate academic research and support data-driven safety policy development within professional cycling.
5. Through ongoing surveillance, objectively evaluate the effectiveness of injury prevention initiatives and strategies to reduce injury.

### Structure of the injury (and illness) surveillance system data model

At its foundation, the model is built around a Core Event Data Set, collected for every identified incident (or Did-Not-Finish/Did-Not-Start (DNF/DNS) case), which captures the essential contextual information needed to classify the event. Following this, medical staff indicate whether the incident resulted in an injury, triggering the relevant reporting pathway.

**Figure 2:**
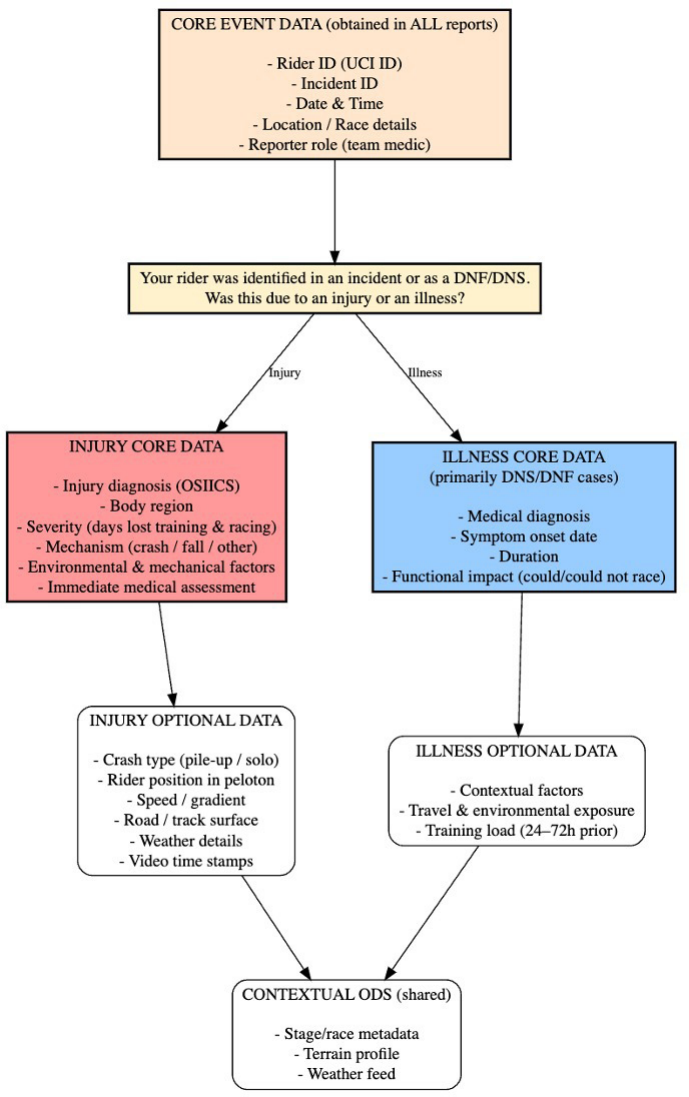
Core data are collected for all injuries (and illnesses), with examples of optional and contextual data providing enhanced detail where required. DNF, Did-Not-Finish; DNS, Did-Not-Start; ODS, Optional Injury Data Set; OSIICS -Orchard Sports Injury and Illness Classification System, UCI, Union Cycliste Internationale.

For injuries, a Core Minimum Data Set is completed, including unique rider and incident identifiers, diagnostic details, body region, mechanism of injury, severity expressed as days lost from training and competition and key environmental or mechanical contributors. The accompanying Optional Injury Data Set allows additional detail to be captured where available, such as crash characteristics, rider position within the peloton, surface and gradient, weather factors and video time stamps that support mechanism verification (figure 2).

For illnesses occurring during grand tour events that result in DNF, a parallel Core Illness Data Set captures the medical diagnosis, symptom onset, duration and functional impact, with an Optional Illness Data Set enabling additional contextual information, such as travel exposures, environmental contributors or recent training load (figure 2).

A final Context Data Set, largely automated, provides race-stage characteristics, terrain and weather feeds and other contextual variables that enhance interpretation across both injury and illness records.

Together, these linked data layers create a scalable, standardised and context-responsive surveillance framework for professional cycling. The structure supports high-quality longitudinal monitoring, enhances comparability events and strengthens the evidence base for targeted injury and illness prevention strategies.

### Data quality assurance and system governance

The proposed injury surveillance system was developed to provide a secure, standardised and interoperable framework for recording injuries and illnesses occurring during UCI World Tour and UCI Women’s World Tour events. The system integrates medical reporting, centralised data storage, secure access management and contextual data analysis within a unified architecture. Development was undertaken by IDLab (Ghent University–imec) in collaboration with Queen’s University and University of Edinburgh researchers and the UCI.

To ensure the validity and completeness of data collected through the surveillance system, a structured quality assurance framework has been embedded in both the technical infrastructure and operational governance processes. All submitted injury and illness records are automatically cross-referenced with race incident data and rider withdrawal logs to verify that medically attended events are captured and correctly classified. Discrepancies—such as race withdrawals without corresponding incident ID entries are flagged for review by the SafeR Case Management Committee during its weekly meetings.

Periodic data validation reports are generated by IDLab and reviewed jointly by the UCI Medical Department and academic partners to identify missing or duplicate entries, incomplete data fields and inconsistencies between incident metadata and clinical reports. Where required, clarification requests are issued to team medical staff to reconcile incomplete or ambiguous cases.

Furthermore, the system incorporates automated integrity checks during data entry, including timestamp verification, duplicate prevention and validation of mandatory fields. Quarterly audits by the SafeR Supervisory Board and the research team ensure adherence to data entry protocols, ethical standards and GDPR compliance.

**Figure 3:**
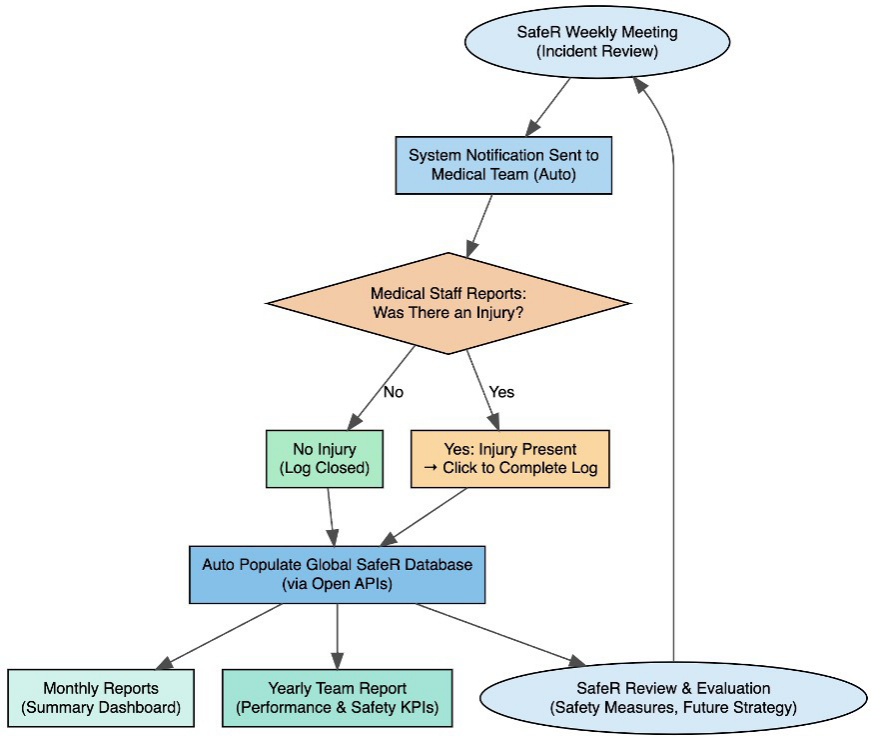
Schematic overview of the injury surveillance system fitting within SafeR structure. Application Programming Interface (APIs), Key Performance Indicators (KPI)

Collectively, these quality assurance measures ensure that the surveillance system maintains a high level of data fidelity, thereby supporting accurate epidemiological analyses and reliable longitudinal monitoring of injury and illness trends across the UCI World Tour and Women’s World Tour (figure 3).

### Injury data definitions and collection

Injury and/or illness data are collected via a bespoke electronic form developed in Qualtrics, which offers high levels of customisability in line with the US Olympic and Paralympic Committee injury surveillance system methods.^41^ The form is accessible on mobile and desktop devices for field use across diverse racial environments and requires no sign-up or sign-in credentials.

The definitions of injury and illness used in this surveillance system were adapted from the 2020 IOC Consensus Statement on injury and illness surveillance and its cycling-specific extension.^5 29^ In agreement with the US Olympic and Paralympic Committee surveillance system definitions, the modification was intentional and undertaken to align with the specific operational objectives of the present platform, namely, to record and analyse only those injuries and illnesses that necessitate clinical evaluation and formal diagnosis by qualified medical personnel (UCI or team-affiliated clinicians) occurring during an event.^41^ This design choice was informed by the practical and regulatory context of elite road cycling, in which the emphasis lies on medically verified health events rather than self-reported or subclinical reports.

Accordingly, an injury is defined as:

‘Tissue damage or other derangement of normal physical function sustained during participation in a UCI World Tour or UCI Women’s World Tour event that requires evaluation by a healthcare provider and results in a clinical diagnosis.’

Similarly, an illness is defined as:

‘A physical health-related complaint or disorder experienced by a rider, resulting in a DNF or DNS not attributable to injury, that requires evaluation by a healthcare provider and results in a clinical diagnosis.’

These definitions ensure that the surveillance system captures clinically substantiated cases with diagnostic confirmation, thereby enhancing the validity, specificity and comparability of data across teams and competitive contexts. Health-related encounters that do not meet these criteria, such as generalised soreness, muscular tightness or other subclinical conditions addressed through recovery or maintenance services (eg, massage therapy or physiotherapy without diagnostic assessment) are classified as performance or recovery encounters and are not incorporated into the formal injury or illness dataset.

**Figure 4:**
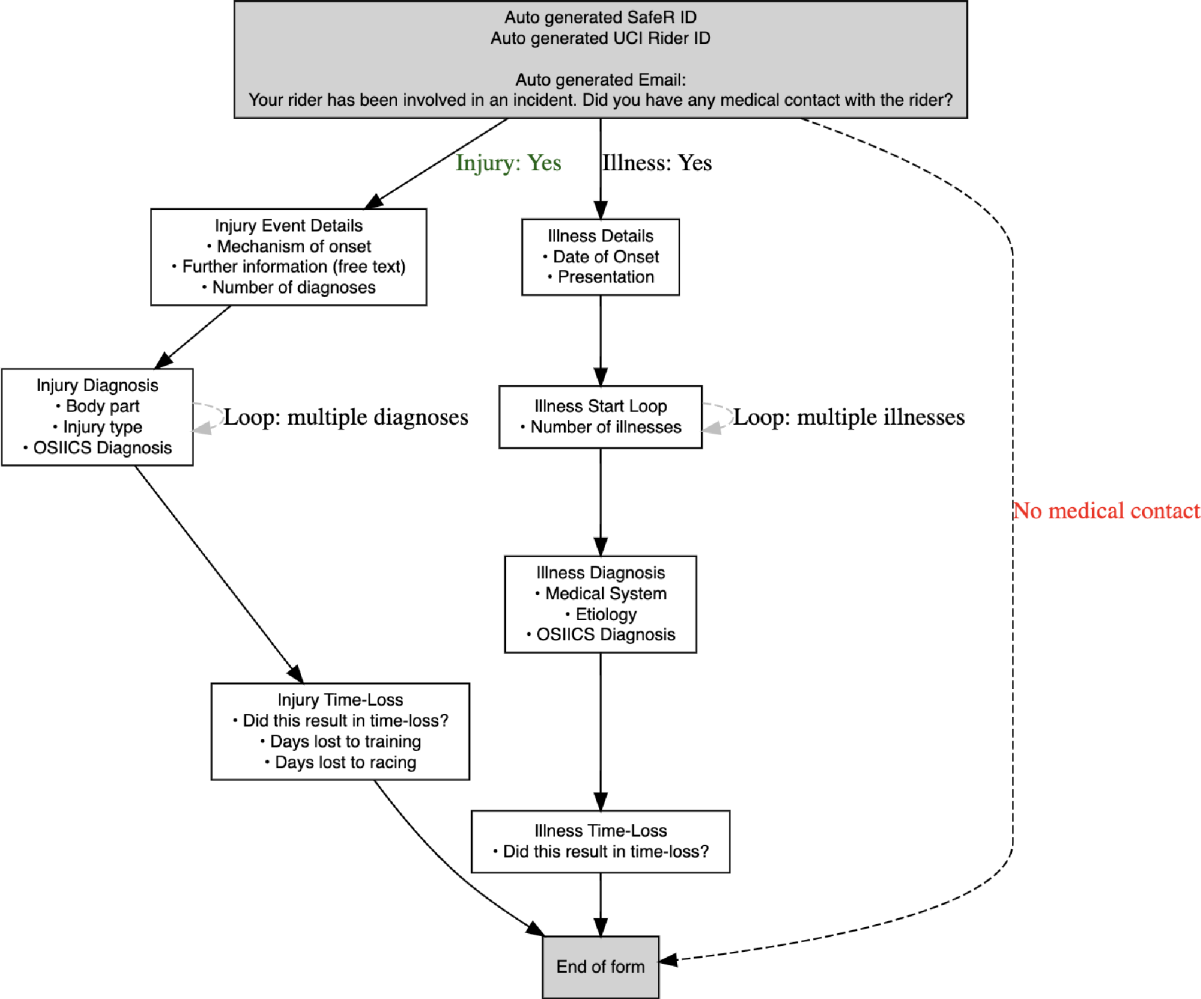
Schematic overview of the injury surveillance system log completed by doctors, which auto-populates the central incident database on completion. OSIICS -Orchard Sports Injury and Illness Classification System, UCI, Union Cycliste Internationale.

Automated features, including logic branching, timestamping and input validation, ensure accuracy and completeness. Athlete data are pseudonymised at the point of entry, with identifiers managed separately by the incident database. A complete overview of the system is presented in figure 4.

### Data architecture, storage and security

The surveillance infrastructure comprises four core components: (1) a digital front-end for medical reporting via Qualtrics, (2) a centralised server environment, (3) a JSON Web Token (JWT)-authenticated API and (4) Analytical and multimedia integration modules. Data flow follows a sequential process encompassing data entry, secure transmission, validation, contextual integration and authorised access for research and reporting (figure 2).

#### Secure data storage infrastructure

All injury data are hosted in Qualtrics on the Queen’s University Belfast Server and at IDLab, Ghent University, within a secure, access-controlled server environment. IDLab server architecture employs Advanced Encryption Standard (AES) with a 256-bit key length (AES-256), firewall segmentation and weekly encrypted off-site backups. Physical and remote access are restricted to authorised personnel via institutional Virtual Private Network and Secure Shell key authentication. Data are validated automatically to identify duplicate, incomplete or inconsistent entries before integration into the central database.

#### API architecture and access management

A JWT-authenticated API enables interaction between the database and external applications, such as the Qualtrics injury log. UCI Database access permissions are role-based, with specific privileges for clinicians (data entry and modification), researchers (read-only access following ethical approval) and system administrators (data governance and maintenance). No unauthorised personnel will have viewable access to this data. All data collected via the Qualtrics platform will be managed in compliance with the GDPR standards. The Qualtrics environment provides encrypted data transmission (Transport Layer Security (TLS) V.1.2 or higher) and secure, access-controlled storage. All data transmissions occur over HTTPS, and transaction logs are maintained for full auditability. Endpoint minimisation and rate limiting further reduce exposure to potential security vulnerabilities.

#### Integration of contextual and race-level data

Each injury report is linked to race metadata via the Incident ID and Rider ID, including event characteristics, environmental conditions and, where available, video footage. This ID network provides a metadata framework that aligns injury reports with race footage, enabling the examination of injury mechanisms, contributing factors and environmental correlates. Future iterations will support integration with telemetry (rider tracking) and commissaire comments to enhance multivariate analyses.

#### Data protection, governance and ethical compliance

The system complies with the GDPR and the UK Data Protection Act (2018). Data are pseudonymised before transmission, and riders provide informed consent through their team-administered contractual agreements with the UCI. As part of the annual licensing and registration process, riders acknowledge that incident, injury and illness data recorded during UCI-sanctioned events may be processed for safety, medical governance and research purposes. All access is logged, monitored and reviewed through a governance framework jointly managed by the UCI and academic partners Queens University Belfast. Research access is contingent on ethical approval from institutional review boards, and annual audits are conducted to review data handling, access controls and compliance practices.

### Implementation and evaluation

The system implementation follows a phased deployment strategy that aligns with the WHO steps (online supplemental material 1).

- Phase 0: Introduction to the system and presentation of the current injury log integration to the heads of medicine of each World Tour Team for feedback (July 2025).
- Phase 1: Internal testing using historical anonymised datasets to evaluate data integrity, functionality and security performance (September 2025).
- Phase 2: Live pilot deployment during the 2026 UCI World Tour season, incorporating mixed-methods evaluation in the first quarter to capture usability and adoption metrics.
- Phase 3: Minor changes depending on feedback, with continued deployment across all participating teams for the 2026 season, supported by ongoing user training and technical assistance.

### Data analysis and reporting

Aggregated data will be analysed descriptively to quantify injury incidence, type and mechanism, expressed per 365 athlete race days. Integration of contextual data (eg, video and telemetry) will support exploratory modelling of risk factors and mechanism-specific analysis. Although the primary purpose of the surveillance system is to monitor and analyse injuries occurring during UCI World Tour and Women’s World Tour events, the platform also captures illnesses when these contribute to a rider’s DNF or DNS in Stage races or Grand Tours, which require medical assessment. Illness data will therefore be reported in parallel with injury findings, acknowledging that the volume of reportable illness is expected to be markedly lower and more event-dependent (ie, DNS). Quarterly and annual summary reports will be provided to the UCI and participating teams, and findings will be disseminated through peer-reviewed publications and conference presentations.

### Training and user support

Comprehensive training will be provided to team medical personnel to ensure accurate use of the surveillance system. In addition, user manuals and instructional video tutorials will be developed to serve as ongoing reference materials. A dedicated support staff member will be available throughout the race season to provide guidance and address any operational issues.

## Discussion

This protocol outlines the development of a secure, centralised injury surveillance system tailored for the UCI World Tour and UCI Women’s World Tour, a road race category with relatively high injury incidence, yet systematic surveillance remains insufficient. The proposed system addresses critical gaps in current practice by integrating secure data management, standardised injury classification and contextual race data in a unified framework aligned with IOC consensus statements.^5 29^

Professional cycling presents unique challenges for injury monitoring due to the dynamic nature of race conditions, the decentralised structure of team medical services and variability in injury definitions. In sports such as football and rugby, injury surveillance systems, such as the UEFA Elite Club Injury Study^40 50^ and the Rugby Injury Surveillance Project,^44^ have consistently been used to inform evidence-based rule changes^45 51 52^ and other injury prevention strategies. While cycling lacks a universally implemented injury reporting standard, this makes the development of targeted injury prevention strategies difficult and the evaluation of their effectiveness in reducing injuries impossible.

This system follows the WHO step-by-step framework for establishing an injury surveillance system, ensuring a structured and systematic approach to design, implementation and governance (figure 1, Appendix 1). For the recording and reporting of injuries and illnesses, the platform adheres to the IOC consensus statement and the cycling-specific extension, providing standardised case definitions and harmonised data collection procedures.^5 29^ Together, these frameworks ensure methodological rigour, support longitudinal data capture and establish a robust foundation for future epidemiological analyses, including crash biomechanics and race-environment interactions.

A key advance of this protocol lies in its robust data security model. Previous attempts at centralised health data collection in sports have often faltered due to concerns around athlete privacy or regulatory non-compliance. To safeguard rider privacy, the system incorporates layered data protection measures, including encrypted storage, role-based access control and pseudonymisation at the point of data entry. In addition to meeting GDPR and UK Data Protection Act (2018) requirements, all research use of the surveillance data will be subject to formal ethical approval from a university institutional review board. Approval will be obtained through Queen’s University Belfast before any analysis of identifiable or pseudonymised data. This ensures that rider health information is processed only within a clearly defined ethical and governance framework, and that athletes retain autonomy over how their data is used.

Importantly, the integration and centralisation of video and sensor data (eg, Avg Speed, Time Static) into medical reporting introduces novel possibilities for understanding injury mechanisms. Additionally, the option to provide free-text information about the incident from the medical staff and the athlete enables the gathering of context specific to the incident.^53 54^ This cross-modal data synthesis is particularly relevant in cycling, where precise causal inference is complex due to the complexity and speed of incidents. The ability to synchronise crash footage with injury epidemiology may enable both qualitative and machine-learning-driven pattern recognition, enhancing both clinical and prevention decision-making.^55^ Over time, this system will generate data to inform injury-prevention efforts for sports medicine clinicians and policymakers. Additionally, it will provide insights into the complex system in which cycling-related injuries occur.^56 57^

### Implementation feasibility

A barrier to injury surveillance in professional sport is organisational reluctance, often stemming from concerns over data ownership, competitive confidentiality and administrative burden. This system’s design addresses these concerns by embedding ease of use into the Qualtrics-based front end and maintaining control over access permissions. Additionally, active collaboration with team doctors and the UCI medical team during the development phase not only fosters stakeholder engagement and contextual alignment but also supports adherence to regulatory requirements. According to UCI regulations, teams must submit requested medical information to the UCI and ensure that the surveillance platform operates within established governance structures.

The rollout strategy allows for refinement after the first quarter, if required, with pilot testing completed before the 2026 season, enabling the identification and resolution of operational challenges. Provision of technical support and user training further facilitates adoption, while embedded feedback mechanisms ensure the system evolves in line with user needs and emerging research priorities.

### Limitations and challenges

Several limitations warrant consideration. First, relying on the team medical staff for data entry introduces variability in reporting rates. While standardised forms mitigate this to an extent, inter-rater variability remains a risk. To mitigate this, regular reminders of incomplete incident logs will be provided before actioning the UCI rule, which can request access to the rider’s medical notes to obtain such information.

Second, while the current system supports the integration of video and metadata, analysing such data requires substantial computational infrastructure and expertise, which may limit short-term utility. The ethical implications of video surveillance, especially in high-impact crashes, also require ongoing oversight and rider input. Future work in developing a framework to standardise consensus on the analysis of video race footage for cycling, in addition to providing a reporting structure that gives options for riders to comment on incidents.^58^

Third, although the infrastructure is designed to support expansion into other cycling disciplines beyond road cycling, contextual adaptations will be necessary to account for the different injury profiles of, for example, track, mountain bike and cyclocross.

## Dissemination and future directions

Findings and methodologies from the project will be disseminated through:

- Annual reports will be made available to the UCI, teams and athlete representatives.
- Peer-reviewed publications and presentations at international sports medicine conferences and UCI congresses.
- Collaborative academic partnerships for secondary data analysis.

Beyond its immediate utility for data collection, this system lays the groundwork for several developments. These include:

- Predictive modelling: Using the accumulated dataset to identify risk profiles based on rider characteristics (number of years professional, rider type (ie, domestic, general classification, sprinter), race types or environmental factors.
- Policy impact: Providing empirical evidence to inform race safety regulations, equipment standards and team support protocols, including evaluation of the effectiveness of these interventions.
- Athlete-centred care: Enabling personalised health monitoring across seasons, particularly for riders recovering from injury or managing chronic/recurring injuries (ie, multiple concussions).

## Patient and public involvement

Patients or members of the public were not involved in the design, conduct, reporting or dissemination plans of this research. The study focuses on developing a technical injury-surveillance infrastructure within elite professional cycling, in collaboration with governing bodies, team medical staff and academic researchers. Opportunities for future athlete and stakeholder involvement in system refinement and evaluation are planned during subsequent implementation phases.

## Conclusion

This injury surveillance system represents a critical step forward in enhancing the safety of professional road cycling. By combining rigorous data security with practical usability and research readiness, it addresses long-standing deficiencies in injury documentation. It opens new avenues for scientific inquiry, protective equipment and evidence-based policy development. Its successful implementation will depend not only on technological robustness but also on continued collaboration between clinicians, researchers, governing bodies, teams and athletes.

## Supplementary material

10.1136/bmjsem-2025-003192 online supplemental file 1

## Data Availability

No data are available.
