# Supplementary material for: Development of a secure, standardised and interoperable surveillance platform for race-related injury and illness data within the UCI men’s and women’s road cycling world tour: a study protocol
Source: BMJ Open Sport Exerc Med. 2026 Feb 19;12(1):e003192. doi: 10.1136/bmjsem-2025-003192 (PMC12927301; doi:10.1136/bmjsem-2025-003192)
Supplement: online supplemental file 1 [file bmjsem-12-1-s001.docx]

**Supplementary Material 1: WHO Steps to the Development of an Injury Surveillance System**

| Step | Description | Status |
| --- | --- | --- |
| 1 | Define purpose and objectives | Completed |
| 2 | Identify target population and settings | Completed |
| 3 | Develop case definitions and classification | Completed |
| 4 | Design data collection methods | Completed |
| 5 | Determine data sources and reporting procedures | Completed |
| 6 | Develop data management and storage protocols | Completed |
| 7 | Establish data quality assurance mechanisms | Completed |
| 8 | Develop analysis, interpretation, and reporting plans | In Progress |
| 9 | Implement ethical and governance frameworks | Pending Under Peer Review |
| 10 | Pilot test and implement the system | Partially Complete |
| 11 | Train users and provide ongoing support | Planned |
| 12 | Monitor, evaluate, and refine the system | Planned |
| 13 | Disseminate findings and promote use of data | Planned |
